# Idiopathic Parkinson’s Disease Unmasked by Valproate-Induced Parkinsonism in a Patient With Vestibular Migraine

**DOI:** 10.7759/cureus.92131

**Published:** 2025-09-12

**Authors:** Teru Kamogashira

**Affiliations:** 1 Department of Otolaryngology, University of Tokyo, Tokyo, JPN

**Keywords:** drug-induced parkinsonism, parkinsonism, parkinson’s disease, valproate, vestibular migraine

## Abstract

We report a case of idiopathic Parkinson’s disease (PD) in a patient with vestibular migraine (VM), initially suspected to have valproate-induced Parkinsonism. Valproate was started at 200 mg/day and titrated to 1,000 mg/day over four months for headache control. Although valproate provided effective headache relief, neurological symptoms, including hand tremor, shoulder stiffness, drooling, and adiadochokinesis, emerged and persisted despite discontinuation of the drug. Subsequent imaging studies, including dopamine transporter-single-photon emission computed tomography and 123I-meta-iodobenzylguanidine myocardial scintigraphy, confirmed the diagnosis of PD by the neurology department. This case highlights the need for careful monitoring of neurological signs during and after valproate therapy in VM patients, as latent PD may be unmasked.

## Introduction

Valproate is commonly used to manage vertigo, dizziness, and headache in patients with vestibular migraine (VM) [1]. While its typical side effects include gastrointestinal issues such as nausea, vomiting, and diarrhea, valproate-induced Parkinsonism is a rare but recognized complication [2,3]. Parkinsonism symptoms typically develop insidiously after months to years of valproate therapy, often despite therapeutic serum levels, and can closely mimic idiopathic Parkinson’s disease (PD), causing diagnostic confusion [4-6]. In some cases, dopaminergic therapy provided partial benefit or induced dyskinesia, and valproate likely unmasked latent PD in susceptible individuals. Importantly, discontinuation of valproate consistently led to clinical improvement, with recovery occurring over days to months. We present a case where a patient with VM developed persistent Parkinsonism after valproate therapy, ultimately diagnosed as idiopathic PD.

## Case presentation

A 58-year-old male with a long-standing history of headaches since his 20s presented with persistent floating dizziness and rightward gait deviation for the past year, which had gradually worsened. He also experienced episodic dizziness with headache about three times per week, without associated aural symptoms. He had been self-medicating with over-the-counter analgesics (loxoprofen and acetylsalicylic acid with ibuprofen) and had previously used various medications, including methylcobalamin, adenosine triphosphate, medazepam, escitalopram, clonazepam, and Hangebyakujutsutemmato (Japanese traditional herbal medicine (Kampo)).

Vestibular testing showed no gaze-evoked and positional nystagmus. Pure tone audiometry showed normal thresholds, averaging (500, 1000, and 2,000 Hz) 16.7 dB HL in the right ear and 18.3 dB HL in the left. Posturography recorded total trajectory lengths of 107.43 cm (eyes open) and 133.20 cm (eyes closed). Electronystagmography showed normal smooth pursuit and good optokinetic nystagmus, with no gaze-evoked nystagmus. Ice water caloric testing revealed slow phase velocities of 4.6 degrees/second on the right and 16.0 degrees/second on the left, with 56.3% visual suppression. Canal paresis calculated using the Jongkees’ formula [7] was 55.3%, indicating right-sided peripheral vestibular dysfunction. The cervical vestibular evoked myogenic potential testing showed no response bilaterally, whereas ocular vestibular evoked myogenic potential responses were bilaterally normal. Psychological assessments revealed moderate stress and emotional predisposition: Dizziness Handicap Inventory [8]: 72; State-Trait Anxiety Inventory [9]: 46 (state-anxiety), 44 (trait-anxiety); Self-rating Depression Scale (SDS) [10]: 51; Perceived Stress Scale [11]: 14. The SDS was used after payment of a license fee corresponding to the cost of the questionnaire sheets. No license is required for other questionnaires. MRI showed no abnormalities. Differential diagnosis included probable VM, psychogenic vertigo, and right peripheral vestibular dysfunction.

Initial treatment with vestibular rehabilitation, lomerizine, and Ryokeijutsukanto (Kampo) failed to improve symptoms of dizziness with headaches. Alternative preventive medications for headache (Goshuyuto (Kampo), propranolol, eperisone, and tofisopam) were also ineffective. Valproate was started at 200 mg/day and gradually increased to 1,000 mg/day over four months, which provided better headache control. However, new symptoms appeared after one month at the high dose: hand tremor, shoulder stiffness, drooling, and adiadochokinesis. These were attributed to valproate, and the dose was tapered over four months. Propranolol was introduced for headache control instead of valproate.

**Figure 1:**
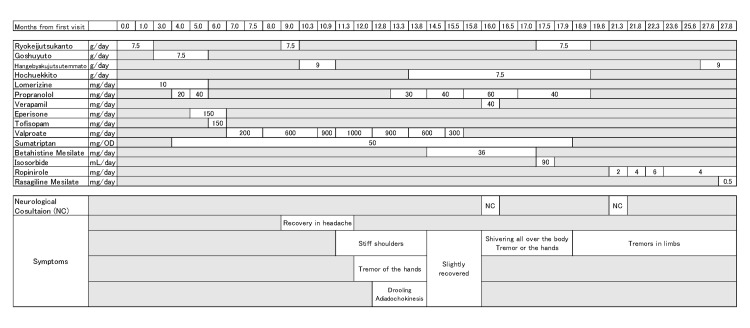
Timetable of the prescription of this case.

The prescriptions over time are shown in Figure 1. Neurological abnormalities persisted one month after discontinuing valproate, prompting referral to the neurology department for further evaluation; however, he was advised only to continue follow-up. Although dizziness with headache was slightly controlled by propranolol, after six months, the neurological symptoms, including tremor of both upper limbs, impaired movement of the left upper limb, and generalized tremor, had not improved, leading to a second referral to the neurology department.

**Figure 2:**
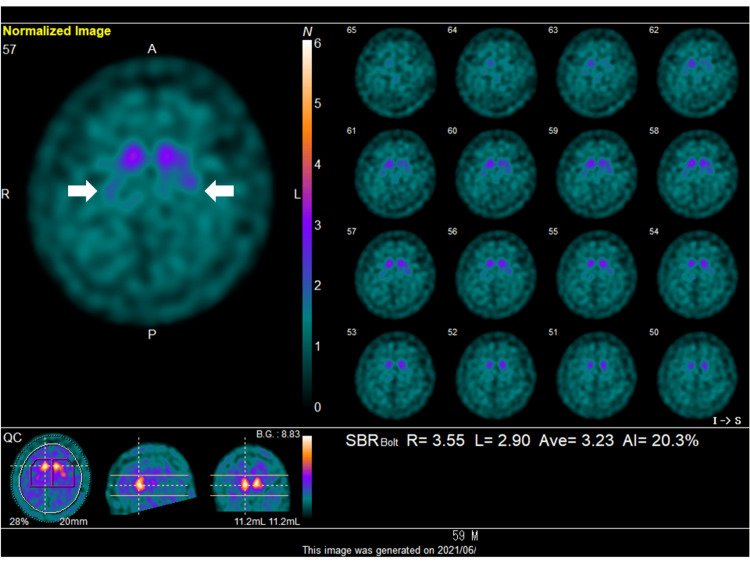
18F-FP-CIT imaging showing reduced DaT uptake in the right striatum consistent with pre-synaptic dopaminergic deficit. White arrows show focally reduced uptake in the bilateral putamen. 18F-FP-CIT = 18F-N-(3-fluoropropyl)-2β-carboxymethoxy-3β-(4-iodophenyl) nortropane; DaT = dopamine transporter

He underwent a dopamine transporter (DaT) scan using single-photon emission computed tomography (SPECT), which demonstrated reduced DaT binding capacity in both the left and right striatum, with a slight predominance on the left. The specific binding ratio was 3.55 on the right and 2.90 on the left, and the age-corrected Z-scores were -3.17 (right) and -4.07 (left), confirming a bilateral decrease in DaT binding capacity (Figure 2). A trial of levodopa at 100 mg/day yielded minimal clinical improvement in symptom control.

**Figure 3:**
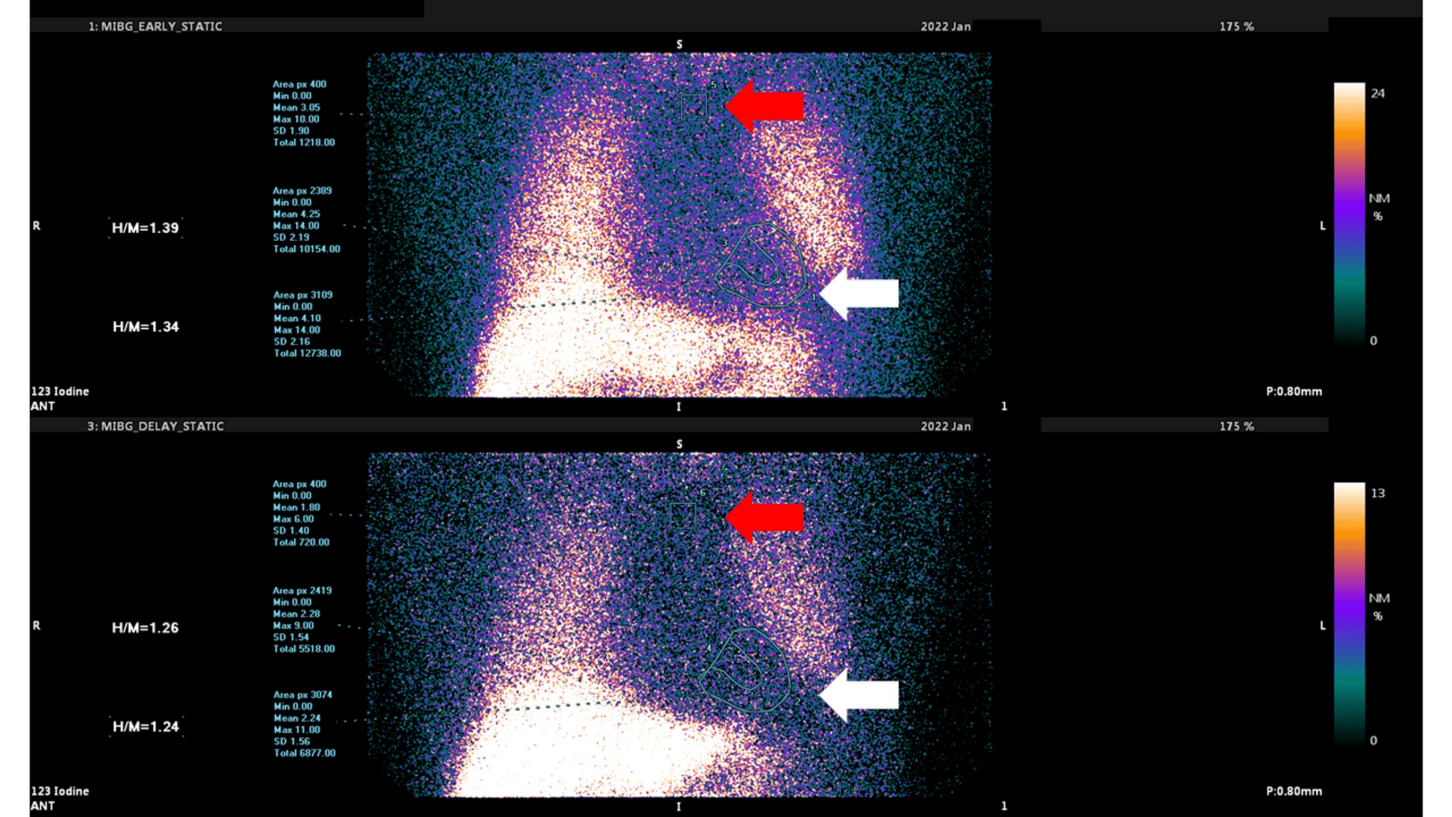
The early H/M ratio using 123I-MIBG myocardial scintigraphy. The heart (white arrows)/mediastinum (red arrows) (H/M) ratio was decreased in both the early (upper) and late (lower) images. H/M = heart-to-mediastinum; 123I-MIBG = 123I-meta-iodobenzylguanidine

Based on the imaging findings, he was diagnosed with PD and started on ropinirole at 2 mg/day, which was later increased to 4 mg/day. Because drooling persisted, rasagiline mesylate 0.5 mg/day was added for symptom control. An additional test with 123I-meta-iodobenzylguanidine (123I-MIBG) myocardial scintigraphy showed a reduced early heart-to-mediastinum ratio of 1.39, below the normal range, further supporting the PD diagnosis (Figure 3). After three months of treatment for PD, dizziness symptoms were partially controlled with Hangebyakujutsutemmato (Kampo).

## Discussion

Migraine and vertigo are among the most common health concerns in the general population. VM is recognized as a distinct diagnostic entity by the Barany Society and the International Headache Society [12], and is one of the common vestibular disorders in the general population, with a reported lifetime prevalence of 1% and one-year prevalence of 0.9% [13]. Various medications are used in VM prophylaxis, including β-blockers, calcium channel blockers, anticonvulsants, and antidepressants [14,15]. The choice of treatment should be guided by side effect profiles and patient comorbidities. In this case, β-blockers were initially prescribed because previously used antidepressants were ineffective. The patient noted significant improvement in headache symptoms with valproate compared to earlier treatments and requested a dose increase.

Valproate is associated with multiple adverse effects, including weight gain, hair loss, bleeding or bruising, tremor, hyperammonemia with encephalopathy due to liver failure, and pancreatitis [16]. Valproate is also used to manage dopamine dysregulation syndrome and impulse-control disorders in PD [17,18]. Valproate-induced Parkinsonism typically emerges after more than one year of medication and is usually reversible, with symptoms resolving within a few months [5,19], although some cases later prove to be idiopathic PD [5]. Reported frequencies of valproate-induced Parkinsonism range widely from 1.4% to 75% [20], reflecting heterogeneity in clinical presentation, age at onset, valproate dose, comorbidities, and imaging findings [16,21]. A psychogeriatric background may also increase susceptibility to valproate-induced Parkinsonism [2]. Long-term maintenance therapy of valproate over one year, generally at doses above 500 mg/day (range 300-2,000 mg/day), with the administered dose in this case being consistent with this range, appears to be the main risk factor [2]. Serum valproate concentrations do not differ significantly between symptomatic and asymptomatic patients and are not reliable for predicting adverse effects [20]. These observations highlight the importance of clinical vigilance when diagnosing valproate-induced Parkinsonism in older patients, particularly those with comorbid neurodegenerative conditions or concomitant antipsychotic use.

In this case, Parkinsonism did not improve after discontinuing valproate for several months, whereas the initial neurology consultation did not diagnose PD. The onset of Parkinsonism after only four months of valproate therapy was shorter than typically reported, suggesting that valproate may have unmasked latent PD. Symptoms persisted despite discontinuation, complicating the diagnosis. Unlike valproate-induced Parkinsonism, which usually shows normal SPECT scans [22], this patient demonstrated abnormal findings, which were decisive in confirming PD. 123I-MIBG myocardial scintigraphy, also useful for differentiating valproate-induced Parkinsonism from PD [23], was abnormal in this case. Although the reported association between migraine and PD is low [24], some studies suggest shared vulnerability factors [25], and drug-induced Parkinsonism has been identified as a predictor of idiopathic PD [26]. These observations emphasize the need for routine neurological examinations at each follow-up visit in patients on long-term valproate therapy. Most prior reports of valproate-induced Parkinsonian symptoms did not include nuclear scans documenting a presynaptic dopaminergic deficit, possibly because the clinical course of valproate-induced Parkinsonism is generally favorable, and nuclear scans were not considered necessary. Future clinical studies of drug-induced Parkinsonism should further assess the role and significance of nuclear scans.

## Conclusions

In patients with VM receiving valproate for headache control, vigilant neurological monitoring is essential. Parkinsonism may indicate drug-induced adverse effects but can also signal the unmasking of idiopathic PD.
